# Analysis of the UK recommendations on obesity based on a proposed implementation framework

**DOI:** 10.1186/1471-2458-10-17

**Published:** 2010-01-15

**Authors:** Amudha S Poobalan, Lorna S Aucott, Sheraz Ahmed, W Cairns S Smith

**Affiliations:** 1 Section of Population Health, Division of Applied Health Sciences, University of Aberdeen, Medical School, Polwarth Building, Foresterhill, Aberdeen AB25 2ZD, UK; 2 Department of Paediatrics and Child Health, Aga Khan University Hospital, Stadium Road, Karachi 74200, Pakistan

## Abstract

**Background:**

There is considerable expertise in the obesity field in identifying, appraising, and synthesising evidence to develop guidelines and recommendations for policy and practice. The recommendations, while based on evidence, are not formulated in a way that readily leads to implementation. This paper analyses the recent UK recommendations on obesity using a proposed implementation framework.

**Methods:**

Two bibliographic databases (Medline and Embase) and various health related and government websites were systematically searched for obesity recommendations published between 1996 and 2007. All the documents published on recommendations for either prevention or treatment of obesity in the UK were assessed. A proposed implementation framework was developed for the purpose of this review. All the UK recommendations were critically appraised and results summarised according to the criteria used within the framework. Cross-country applicability of the proposed framework was assessed using the Swedish policy recommendations on obesity.

**Results:**

Most recommendations on obesity while demonstrating their basis in evidence, fail to meet the implementation standards. They tend to be non-specific in identifying who is responsible for implementation and monitoring, and often no timescale is indicated. The costs of implementation are rarely estimated and those responsible for such funding are not specified. There are some notable exemptions to the general pattern emanating from more operational and locally based groups. The Swedish policy details 79 proposals with responsibility clearly identified and costs are presented for 20 of them. This policy satisfied most of the framework criteria but failed to give details on evaluation, monitoring and the timeframe for implementation.

**Conclusions:**

Public health has developed skills in appraising evidence and formulating recommendations based on appropriate evidence but these are often not implemented. Different skills are required to translate these recommendations into actions. Public health clearly needs to develop the implementation skills to a level comparable to the ability to synthesise evidence.

## Background

The UK adult overweight/obesity prevalence has increased steadily in the past three decades [1-3], despite targets set by the government [4] to reduce obesity levels. A review conducted by National Audit Office (NAO) in 1996 [5] showed no evidence of reduction. The Health Survey for England [6] in 2005 reported two thirds of adults and a third of children as overweight/obese. The recent obesity Foresight document [7] suggests that if current trends continue that by 2015, 36% of males and 28% of females will be obese, increasing to 60% and 50% respectively by 2050. This increase in obesity has consequences for individuals with increased risk of co-morbidities and costs, and for society with the current total cost (including NHS) at £7 billion rising to £50 billion per year by 2050 [7].

Systematic reviews and reviews of reviews [8] have investigated the evidence on prevention and treatment of obesity. These give various recommendations from which policies and strategies have been published with the common aim to reduce the rise in obesity. The aim of this assessment is to critically appraise all published UK obesity recommendations (1996-2007) for implementation criteria using a proposed implementation framework. An additional aim is to assess the cross-country applicability of the developed framework using the Swedish action plan for healthy dietary habits and increased physical activity [9]. This document has been identified as one of the most detailed documents on obesity policies [10] and provides an opportunity to evaluate the framework.

## Methods

An initial scoping exercise was conducted to identify any implementation framework to assess guidelines on obesity. One framework was identified for monitoring and evaluating implementation of the global strategy on diet, physical activity and health published by the WHO in 2008 [11]. This framework suggested that process, outcome and output indicators should be identified by each member state. The literature was also searched for recurrent themes within various recommendations that were relevant to implementation. The proposed framework with critical items was developed based on these recurrent common themes which were: specificity of the target population, responsibility for implementation, monitoring, evaluation, time frame, priorities and cost estimation.

The electronic bibliographic databases, Medline and Embase, were then systematically searched for articles published from 1996 to December 2007. Mesh terms and key words for 'obesity', 'obesity guidelines', 'recommendations' were combined using Boolean operators to identify the relevant articles and reports. The search strategy used in Medline is detailed in the additional file, which was modified for use in Embase (see Additional file 1). A structured search of the internet was undertaken to identify the other guidelines and recommendations not indexed in the electronic bibliographic databases. The sources accessed were Science Direct, Blackwell Synergy, National Electronic Library for Health (Guidelines Finder), University of York Centre for Reviews and Dissemination, Public Health Electronic Library, The National Electronic Library for Health, Scottish Intercollegiate Guidelines Network (SIGN), The National Institute of Clinical Excellence (NICE), Health Development Agency (HDA), Department of Health (DoH), and The Stationery Office site. The key words used for the website searches were 'obesity', 'guidelines' and 'recommendations'. All the identified abstracts were scanned by two reviewers and full texts of potentially eligible documents were obtained and assessed according to the inclusion criteria.

All the included UK recommendations were appraised using the proposed framework. The relevant details were extracted from all the documents included. The assessment of the obesity recommendation documents are summarised according to this framework. The Swedish action plan for healthy dietary habits and increased physical activity [9,10] was critically appraised using the same criteria to assess the cross-country applicability of the developed framework.

## Results

**Figure 1:**
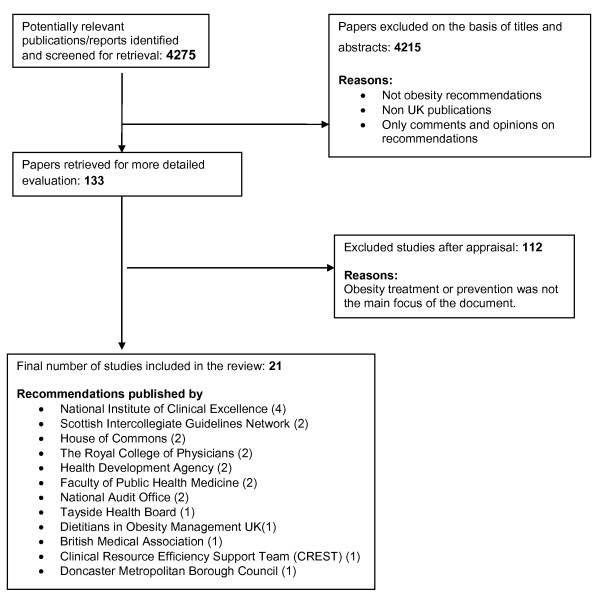
**Selection process of the review**. Flow diagram of the selection process of the review for the appraisal.

The systematic search identified 4275 abstracts, of which 133 were potentially eligible. The full texts of these were critically appraised and 21 articles were included in the review. The results of the literature search and the selection process are presented in Figure 1.

### Key recommendations for obesity identified in selected UK reports

The reports identified key nutritional recommendations. These were to replace energy dense snacks and drinks with healthier alternatives from vending machines in school and fast food outlets [12-14]; to train teachers in healthy food advice and physical activity [12]; to shift consumer demand from high fat, high calorie diets to healthier alternatives [12,15] with the Government and Food Standard Agency (FSA) working together; to simplify food labelling for easy interpretation by the general population [16]; to ban marketing of unhealthy foods targeting children [17,18]; and to provide healthy diet and physical activity advice to pregnant and/or breast feeding women to promote weight control [14,15].

The reports identified key recommendations for physical activity. These were that schools and local authorities should improve physical activity levels by allocating ≥ 3 hours per week for physical activity among school children; make safer pedestrian routes [12-14]; provide information about pedometers for all age groups [12,14] and to consider single sex physical education classes to improve participation of girls and ethnic minority groups [12-14].

The recommendations for obesity management were that physicians should maintain databases for patients at risk of developing obesity [19] and for those receiving obesity treatment (drugs and surgery) [20-22]; that the Government should provide sufficient funds for the NHS for at least one specialist primary care obesity clinic within each Primary Care Trust area and to expand obesity services in secondary care to include bariatric surgery for morbidly obese people [12,23]; that easy access to specialist treatment for obese children and young people should be provided [24] and funds should be made available for doctors and nurses to train in obesity management [15,23].

These reports recommended that the Government should initiate a health education campaign specifically for tackling obesity [12,15]. Guidelines for drugs and obesity management should be constantly evaluated [25] with information about effectiveness of obesity treatment and preventative interventions being disseminated to appropriate health care professionals [26].

### Analysis of UK obesity recommendations using the Implementation Framework

The 21 selected reports were analysed using the proposed implementation framework based on 7 criteria (see Additional file 2). The findings are summarized in Table 1. All 21 studies [12-32] clearly define the target population and prioritise in terms of either prevention and/or treatment. Sub-groups of the community vulnerable to obesity are specifically targeted within recommendations. The organisations responsible for implementation [12-26,28-32] was considered by 20 of the studies. The Government, Department of Health, Cabinet Task Force, NHS and physicians were identified as having responsibility for monitoring of implementation, but 5 out of the 21 articles did not report on how the implementation progress should be monitored or evaluated. Achieving set milestones, conducting regular audits and maintaining databases on progress were tools suggested for monitoring and evaluating the progress of implementation. Although stated, there was no evidence of ownership of these published recommendations.

**Table 1:** Results of the analysis of recommendations. Analysis of essential elements within the recommendations using the Implementation framework

Implementation framework elements	Number out of 21 set of recommendations	Details
Target population	21 out of 21 14 = Both adults and children;[12-16,19,23,25-29,31,32] 4 = Children and young adults (up to 18 years) only;[17,18,24,30] 3 = Obese adults [20-22]	Children and adults, obese adults, children of obese parents and with a family history, people with diabetes and coronary heart disease, low income groups, pregnant women, smokers, disabled people and ethnic groups

Responsible agency	20 out of 21[12-26,28-32]	Government, Department of Health for overall development and implementation of strategies NHS for management within NHS and training of GPs and nurses GPs for implementation of clinical guidelines and maintaining the audit for compliance Local authorities for local implementation of recommendations (i.e safe routes) Food Standard Agency for Nutrition Department of Sports, Sports England and Sports Scotland for Physical activity

Monitoring and Evaluation	16 out of 21[12-16,19-23,25,26,28,30-32]	One third of the articles did not report on how the implementation progress would be monitored or evaluated. The Government, Department of Health, cabinet task force, NHS and physicians were implied for the monitoring of implementation. Achieving the set milestones, conducting regular audits and maintaining databases on progress were few of the tools suggested for monitoring progress of implementation.

Time-frame for the implementation	4 out of 21[13,14,23,31]	Very few set out specific time-frame for implementation. Two studies[12,24] stressed implementation was urgent and some studies anticipated problems in implementing the recommendations

Prioritisation	21 out of 21 4 had treatment as priority [20-23] 2 had treatment and prevention[27,32] 15 had prevention only as priority [12-19,24-26,28-31]	Although the recommendations was separated out broadly into 'Treatment' and 'Prevention', in many there was a long list of recommendations without any priority for specific components

Cost and resources	7 out of 21[14,20-23,28,32]	Seven studies estimated the costs to the NHS for implementation of their recommendations. 11 did not mention cost or funding and the rest only recognised cost as an issue for successful implementation

Only four reports considered an implementation timeframe [13,14,23,31]. The report by the Faculty of Public Health [13] set the time for achieving targets to be within 3 years of their report with goals set for the 1^st^, 2^nd ^and 3^rd ^year whereas the Tayside report [14] set a 10 year timeframe with goals set at the 1^st ^and 5^th ^year. The other two reports mentioning timeframes gave no details. Two reports by NICE [20,21] predicted the uncertainty in implementation due to lack of expertise and resources plus training of doctors. Two other reports [12,24] merely stated that the implementation of recommendations was urgent.

Seven reports gave estimated implementation costs [14,20-23,28,32]. NICE gave NHS estimated costs for orlistat, sibutramine and bariatric surgery recommendations [15-17]. The Tayside local strategy for obesity report [14] gave costing for the extension of their weight management service to all Tayside GP practices, child obesity services and their food "dudes" programme [14]. One report [28] identified resources along with skills required for interventions. Of the remaining, eleven gave no costing, two others [13,19] suggested that their recommendations should be implemented after considering the available resources and the "Toolkit for obesity" by the Public Health Faculty [31] recommended that the NICE costing templates [32] for adult and childhood obesity management should be used.

### Cross-country applicability of the developed framework

The Swedish action plan [9] has been identified as one of the most detailed documents [10] addressing obesity as part of the action plan for healthy dietary habits and increased physical activity. It has 79 proposals (called measures) in 12 specified policy areas (see Additional file 3) with detailed descriptions of the justification for each measure. It clearly identifies the people responsible for implementing all the 79 proposals highlighted. Only 20 out of the 79 proposals gave cost estimates, with one proposal indicating the split between development and implementation. However, the action plan did not provide adequate information in terms of monitoring, evaluation and time frames. Some of the proposals highlight the importance of evaluation but details of how this might be achieved or who would be responsible for the evaluation was not clear. The breakdown of the costing in 4 of the proposals gave an indication of time frame (e.g. EUR 8.5 million over 7 yr period or EUR 210.000 per year for 3 years and EUR 53.000 per year for 5 years), but was otherwise not clearly stated. Within the proposals, gaps and limitations which need to be addressed were identified, for example the lack of health information to ethnic minorities, lack of evaluation of organisational measures, and shortage of intervention research in Sweden.

## Discussion

### Main findings of this review

This critical appraisal of obesity prevention/treatment recommendations in the UK using implementation criteria indicates that some aspects such as priorities and target populations are generally well laid out. However, important factors such as timeframes and cost estimations are not adequately addressed. The responsible organisations are often identified but actual ownership of the recommendations is unclear. Treatment recommendations for drugs and surgery were more specific with projections of cost and future eligible populations. However, prevention recommendations tended to lack clarity for timeframes and costings.

### What is known and what this review adds

There is considerable expertise in the process of identifying, critically appraising, and synthesising the evidence to develop guidelines and recommendations for obesity policy and practice. However, there are indications that these recommendations are failing to be implemented despite being evidence based, which may be due to their formulation and presentation.

This assessment is the first to systematically appraise recommendations for obesity treatment/prevention in terms of the criteria for their implementation. All the recommendations within UK and one action plan from Sweden were appraised using an implementation framework. Another framework recently proposed by Sacks *et al *[33] has analysis grids for a comprehensive policy approach to reducing obesity hence identify areas for obesity policy action. Our review leads on from this by proposing criteria within such policies to be addressed for easier implementation.

Recommendations need to be framed in a manner to facilitate their implementation and this includes targeting, ownership, monitoring and evaluation, time frame and resource implications. This approach is generalisable and can be used to assess other strategy documents and their recommendations. It is worth noting that evidence based guidelines/action plans do not always give the essential elements for implementation at the initial stage but may be extended as formal implementation plans at a later date.

The NHS Modernisation Agency [34] with 24 Primary Care Trusts (PCTs) conducted a review to identify obesity strategies developed by the Trusts as a response to recommendations issued by the Faculty of Public Health [13]. This review found that the Trusts were at the early stages of development and implementation, and highlighted the evidence of current best practices by various Trusts. Since this review, two strategies have been published in England [35] and Scotland [36] which move away from focusing on the individual and instead consider broader holistic integrated approaches to obesity prevention such as healthy lifestyle adoption at all levels of society, but these still do not address the issues if implementation highlighted in this paper. The Swedish action plan identified as one of the most complete documents [9] provides detailed descriptions of 79 proposals and addresses most of the criteria identified in this framework but it does not address the issues of monitoring, evaluation or the setting of time frames. The essential elements identified in this proposed framework encompass issues at the level of recommendation/guideline formation that will facilitate implementation. Successful implementation of guidelines (in whole or in part) will result in various interventions being developed which can be assessed using a Health Impact Assessment [37] which reflects some of the broader issues covered by the proposed framework.

The literature search used a comprehensive strategy but many of the recommendation documents were not electronically indexed in databases and available only on websites. Efforts were made to identify all documents from various sources but recommendations by various groups, charities and local authorities may not be readily in the public domain.

The implementation framework was developed through a scoping exercise and was based on the recurring themes within guidelines and may require modification in light of experience with its use. The proposed framework thus provides a first step in assessing the obesity guidelines to emphasise the importance of addressing the essential elements contained within them for successful implementation.

## Conclusion

Obesity recommendations in UK clearly define the target population and are well prioritized in terms of either prevention and/or treatment. Sub-groups of the community vulnerable to obesity are specifically targeted within recommendations with most identifying the organisations responsible for implementation. However, for recommendations to be successfully implemented, it is essential that they also have clear timeframes, costings and identify ownership, training and coordination within local organisations. Clinicians and academics involved in producing recommendations and policies should consult public health professionals who are more familiar with actual implementation of the proposed actions to ensure that their proposals are realistic for successful implementation. The proposed framework could be used as a basis and adapted for wider use in other countries, for other topics and for different target groups. Every effort should be taken to formulate evidence based recommendations that facilitate their effective implementation in view of the rapidly increasing obesity epidemic.

## Competing interests

The authors declare that they have no competing interests.

## Authors' contributions

The study was designed and planned by WCS. AP and SA carried out the systematic review. LA, AP and WCS contributed to the development of the implementation framework. AP and SA drafted the manuscript with the contribution from all authors. All authors read and approved the final manuscript.

## Pre-publication history

The pre-publication history for this paper can be accessed here:

http://www.biomedcentral.com/1471-2458/10/17/prepub

## Supplementary Material

Additional file 1 **Search Strategy**. Search Strategy used in Medline which was modified for the other databases. Click here for file

Additional file 2 **Analysis based on proposed framework**. Analysis of articles on obesity recommendations based on the proposed implementation framework. Click here for file

Additional file 3 **Cross-country applicability of the proposed framework**. Analysis of the Swedish Action Plan based on the proposed framework. Click here for file
