# Supplementary material for: Analysis of the UK recommendations on obesity based on a proposed implementation framework
Source: BMC Public Health. 2010 Jan 15;10:17. doi: 10.1186/1471-2458-10-17 (PMC2821361; doi:10.1186/1471-2458-10-17)
Supplement: Additional file 1 — Search Strategy. Search Strategy used in Medline which was modified for the other databases. [file 1471-2458-10-17-S1.DOC]

**Additional file 1: Search Strategy used in Medline**

1. exp Obesity/

2. overweight$.tw.

3. quetlet$.tw.

4. (weight adj1 change$).tw.

5. (body adj1 mass adj1 index adj3 change$).tw.

6. (weight adj1 los$).tw.

7. or/1-6

8. recommend$.tw.

9. (government adj3 publication$).tw.

10. (guideline$ adj3 adherence$).tw.

11. (practice adj1 guideline$).tw.

12. guideline$.tw.

13. barrier$.tw.

14. facilitate$.tw.

15. implement$.tw.

16. or/8-15

17. prevention$.tw.

18. (primary adj1 prevention$).tw.

19. (treat$ or therap$).tw.

20. or/17-19

21. exp Great Britain

22. United Kingdom.tw

23.UK.tw

24. or/21-23

25. 7 and 16 and 20 and 24
