# Supplementary material for: Analysis of the UK recommendations on obesity based on a proposed implementation framework
Source: BMC Public Health. 2010 Jan 15;10:17. doi: 10.1186/1471-2458-10-17 (PMC2821361; doi:10.1186/1471-2458-10-17)
Supplement: Additional file 2 — Analysis based on proposed framework. Analysis of articles on obesity recommendations based on the proposed implementation framework. [file 1471-2458-10-17-S2.DOC]

# Additional file 2: Analysis based on proposed framework

| **Title of articles** | **Specificity** | Responsibility | **Monitoring** | **Evaluation** | **Time frame** | **Priorities** | **Cost estimation** |
| --- | --- | --- | --- | --- | --- | --- | --- |
| Obesity in Scotland: integrating prevention with weight management[19] | TP: Adults  Children. | NHS  Physicians | General physician | Audit and software | Not specified | Prevention | Costs and benefits should be evaluated before implementation of the initiatives |
| Coronary Heart Disease. Guidance for implementing the preventive aspects of the National Service Framework[28] | TP: Adult  Children  At risk groups | Health Authority, School nurses, PCGs  Primary care level  Primary care teams, GPs, and dieticians | By achieving the set milestones. | Mentioned the outcome of the interventions. | Not provided | Prevention | Resources for each intervention were mentioned |
| Tackling obesity: A toolbox for local partnership action[13] | TP: children and young people, adults and people with tendency to develop weight gain. | Heath authority  PCGs, NHS Health professionals | NHS and its partner agencies | By achieving the set milestones. | Time frame provided. | Prevention | Implementation should be done after considering the available resources. |
| Tackling Obesity in England. A report by National Audit Office[26] | Recommendations were specific in terms of target population | DoH FSA NHS  Department of education, employment | NHS and department of health should set the realistic milestones | Recommends the indicators of progress in reducing the health inequalities. | Not mentioned | Prevention | Not reported |
| Guidance on the use of sibutramine for the treatment of obesity in adults[20] | Age =18-65 years  TP: overweight or obese Adults | Local NHS in England and Wales | Health professionals and hospitals concerned with the management of obesity. | Clinicians should record the treatment plan for monitoring the side effects of drugs. | Implementation will need additional time due to some training of health professionals. | Treatment guidance. | Costs to NHS for implementation were estimated. |
| Guidance on the use of orlistat for the treatment of obesity in adults[21] | Age = 18-75 years.  TP: overweight or obese Adults | Task for local NHS in England and Wales | Health professionals and hospitals. | Databases should be established by hospitals | Implementation will need additional time due to some training of health professionals. | Treatment | Costs to NHS for implementation re estimated. |

| The Ninth Report of the Committee of Public Accounts: Tackling obesity in England[25] | TP: Children and adult population | DoH NICE GPs NHS | By developing the indicators of progress. | Not reported | Not reported | Prevention | Not reported |
| --- | --- | --- | --- | --- | --- | --- | --- |
| Guidance on the use of surgery to aid weight reduction for people with morbid obesity[22] | Age=Above 18 years  TP = Obese Adults | NHS England and Wales | Health professionals and hospitals | Databases should be established by hospitals | No specific time frame | Treatment guidance. | Estimated the costs to NHS and individual patients for this procedure. |
| DOM (UK) s submission of evidence to the Government’s Health Select Committee into Obesity[29] | Children and adults of all ages. | DoH and media | Not reported | Not reported | Not specified | Prevention | Not reported |
| Management of obesity in children and young people[24] | Children and young people up to the age of 18. | Health professionals | Not reported | Not reported | Some recommendations were termed as urgent. | Prevention | Not discussed |
| The management of Obesity and overweight .An analysis of reviews of diet, physical activity and Behavioural approaches[27] | Adult and children of all ages. | Not reported | Not mentioned | Not reported | No time frame provided for implementation. | Treatment and Prevention. | Not discussed |
| Obesity. Third report of session 2003-04[12] | TP: Children, young people and adults. | Government  DoH  FSA  Ofcom  NHS | Government task force will monitor the initiatives. | Not reported | Recommended the government to take action urgently | Prevention. | The Government should estimate the costs. |
| Storing Up Problems: The medical case for a slimmer nation[16] | TP: Children and adults of all ages.  At risk groups | Government FDA NHS Local authorities | Cabinet level committee | By achieving the set milestones. | Not specified | Preventive interventions | Not reported |
| RCP response to 'Choosing Health' overweight and obesity[15] | Mentioned the age group from childhood to old age. | Cabinet level Task force | Cabinet level Task force | By achieving the set milestones | No time frame was given. | Prevention and maintenance of weight | Not reported. |
| Tackling overweight and obesity[14] | For each action, target groups were specified with age range and justification for each recommendation. | Different departments such as local NHS, GPs, Dieticians, etc | Each department will monitor the progress of implementation | Evaluation of progress after 1 year of implementation | Follow up after 1 year for proof of implementation and then after 5-10 years to see whether the implementation was effective | Prevention | Costs were estimated for some  Programmes. |
| Preventing childhood obesity  A report from the BMA Board of Science [17] | Deals with the children | Government  Schools  Healthcare professionals  FSA | Not reported | Not reported | No time frame | Prevention | Not reported. |
| Guidelines for the management of obesity in secondary care[23] | Adults and children | Government  Healthcare professionals | Not reported. | Audit reports | specified | Treatment | Costs are estimated at £2m/year |
| Report of the  Scrutiny of Child Obesity in Doncaster[18] | Children of age 11 years or under | Government  Primary care trusts  Local council  Other local organizations | Not reported. | Not reported. | Not reported. | Prevention | Not reported. |
| Obesity: the prevention, identification, assessment and management of overweight and obesity in adults and children [32] | Adults and children | NHS  PCTs  Food industry  Local authorities  Schools | Each department will monitor the progress of implementation through audit | Audit reports | Not reported. | Prevention and treatment | Costs are estimated for priority recommendations |
| Tackling child obesity – First steps[30] | Children of age 5-10 | Departments of Health (DH); Department for Education and skills; Department for Culture, Media and Sport;  PCTs; SHAs; LSPs | Departments of Health (DH); Department for Education and skills; Department for Culture, Media and Sport | Not reported | Not reported | Prevention | Not reported |
| Lightening the load: Tackling overweight and obesity[31] | Adults and children | NHS  PCTs; Media; Local authorities; commercial slimming clubs; parents and schools | Responsible departments will monitor the progress | Evaluation and monitoring through Health Education Board for Scotland toolbox | Time frame provided | Prevention | Costing template to be used developed by NICE [32] |
