# Supplementary material for: Analysis of the UK recommendations on obesity based on a proposed implementation framework
Source: BMC Public Health. 2010 Jan 15;10:17. doi: 10.1186/1471-2458-10-17 (PMC2821361; doi:10.1186/1471-2458-10-17)
Supplement: Additional file 3 — Cross-country applicability of the proposed framework. Analysis of the Swedish Action Plan based on the proposed framework. [file 1471-2458-10-17-S3.DOC]

# Additional file 3: Cross-country applicability of the proposed framework

| **Title of article** | **Specificity** | Responsibility | **Monitoring** | **Evaluation** | **Time frame** | **Priorities** | **Cost estimation** |
| --- | --- | --- | --- | --- | --- | --- | --- |
| Background material to the action plan for healthy dietary habits and increased physical activity- Stockholm [9]  **79 measures were detailed in 12 policy areas** | Target population:  - Children  - Elderly  - Healthy adults  Policy Area  - Education  - Workplace  -Transport  - Food  - Taxation  - Environment  - Sports  - Health and Medical  Care  - Public Health  - Research  - Housing  -Consumer | Five different agencies were identified  - Central agencies  - County councils  - Non -governmental organisations  -Universities/ Colleges  - County administrative board  Some of the central agencies were National Institute for Working Life; National Board of Housing , Building and Planning; National Institute of Public Health; National Food Administration; Swedish Sports Confederation; Swedish Board of Agriculture; Swedish Consumer Agency | Not given | The details of evaluation for each proposal were not given within each of the measures, although they highlighted the importance of evaluation. | Time frame not specified within each proposal. However, the breakdown of the costing in 4 of the proposals gives an indication of time frame  e.g. EUR 8.5 million over 7yr period or  EUR 210.000 per year for 3 years and EUR 53.000 per year for 5 years | All 79 measures/motivation were described in detail within the policy areas | 20 Proposals out of 79 had the cost estimated/ stipulated.  Some had a split for development and implementation |
